# Thromboelastographic Results and Hypercoagulability Syndrome in Patients With Coronavirus Disease 2019 Who Are Critically Ill

**DOI:** 10.1001/jamanetworkopen.2020.11192

**Published:** 2020-06-05

**Authors:** Jared Robert Mortus, Stephen E. Manek, Lisa Suzanne Brubaker, Michele Loor, Miguel Angel Cruz, Barbara W. Trautner, Todd K. Rosengart

**Affiliations:** 1 Michael E. DeBakey Department of Surgery, Baylor College of Medicine, Houston, Texas; 2 Center for Translational Research on Inflammatory Diseases, Michael E. DeBakey VA Medical Center, Houston, Texas; 3 Department of Medicine, Baylor College of Medicine, Houston, Texas; 4 Center for Innovations in Quality, Effectiveness, and Safety, Michael E. DeBakey VA Medical Center, Houston, Texas; 5 Section of Health Services Research, Departments of Medicine and Surgery, Baylor College of Medicine, Houston, Texas

## Abstract

This cohort study examines the association of thromboelastographic results with hypercoagulability among patients with coronavirus disease 2019 who have been admitted to an intensive care unit.

## Introduction

Severe acute respiratory syndrome coronavirus 2 (SAR-CoV-2) is responsible for the coronavirus disease 2019 (COVID-19) pandemic that has caused approximately 300 000 deaths globally. Disseminated intravascular coagulopathy and other COVID-19–associated coagulopathies occur among patients with severe SARS-CoV-2 infections.^1^ Potentially lethal hypercoagulability is an unusual, poorly defined COVID-19–associated coagulopathy presentation.^2,3^ We found that more than half of patients admitted to the intensive care unit (ICU) of Baylor St Luke’s Medical Center developed clinically significant thromboses that were associated with hypercoagulable thromboelastographic (TEG) parameters alone.

## Methods

This cohort study was approved by the Baylor College of Medicine institutional review board with a waiver of informed consent granted because this was a retrospective electronic health record review of data collected for clinical purposes. The cohort included all patients admitted to the ICU of Baylor St. Luke's Medical Center from March 15 to April 9, 2020, with SARS-CoV-2 infection confirmed by reverse transcription–polymerase chain reaction test of nasopharyngeal swab. This study is reported following Strengthening the Reporting of Observational Studies in Epidemiology (STROBE) reporting guidelines for a cohort study.

All patients received standard deep vein thrombosis chemoprophylaxis on ICU admission and therapeutic anticoagulation (heparin infusion or enoxaparin [2 mg/kg/d]) for thrombotic complications. All patients underwent TEG and TEG with heparinase correction on ICU admission. Hypercoagulability was defined as elevated fibrinogen activity greater than a 73° angle or maximum amplitude (MA) more than 65 mm on TEG with heparinase correction.

Group differences were analyzed using Fisher exact test. Analyses were conducted using SAS statistical software version 9.4 (SAS Institute). *P* values were 2-sided, and statistical significance was set at .05. Data were analyzed from March 21 to April 14, 2020.

## Results

This cohort study included 21 patients (mean [SD] age, 68 [11] years [range, 50-89 years]; 12 [57%] men). Among these patients, 20 (95%) had comorbidities, with a mean (SD) of 3 (2) comorbidities each (range, 1-7 comorbidities each). Mean (SD) follow-up was 11 (4) days. Regrading thromboembolism risk, 4 patients (19%) had atrial fibrillation, a history of malignant tumors, or chronic kidney disease. Four patients (19%) required extracorporeal membrane oxygenation, and 18 patients (86%) required renal replacement therapy. There were 2 mortalities (10%), both occurring as pulseless electrical activity after acute-onset pulmonary hypertension.

Cohort mean international normalized ratio (INR), partial thromboplastin, and platelet levels were within reference ranges, but fibrinogen and dimerized plasmin fragment D levels were elevated (Table 1). A total of 19 patients (90%) demonstrated hypercoagulable TEG, including 14 patients (74%) with hypercoagulable TEG as defined by fibrinogen activity and MA criteria and 5 patients (26%) with hypercoagulable TEG as defined by MA criteria alone. There were 13 patients (62%) who demonstrated clinical evidence of thrombotic events, with a total of 46 events recorded and a range of 1 to 8 events per patient. All but 1 of these patients presented with arterial, central venous, or dialysis catheter or filter thromboses (Table 1). These patients received therapeutic anticoagulation a mean (SD) of 6 (5) days after ICU admission (range, 1-18 days).

**Table 1.** Patient Coagulation Parameters and Thrombotic Complications

Parameter	Mean (SD)^a^	Reference range
Prothrombin time, s	14.8 (2.4)	11.9-14.2
INR	1.2 (0.2)	≤5.9
Partial thromboplastin time, s	36 (8.0)	22.5-36.0
Platelet count, ×10^3^/µL	210 (100)	150-450
Fibrinogen level, mg/dL	740 (240)	225-434
D-dimer, μg/mL	8.3 (7.0)	<0.5
Thromboelastography		
R value, min	10 (11)	4-7
Fibrinogen activity angle, °	60 (23)	61-73
Maximum amplitude, mm	67 (17)	55-65
LY30, %	0.9 (1.8)	0-5
Thromboelastography with heparinase correction^b^		
R value, min	6.0 (2.7)	4-7
Fibrinogen activity angle, °	73 (10)	61-73
Maximum amplitude, mm	74 (10)	55-65
LY30, %	2.1 (3.7)	0-5
Patients with thrombotic complications, No. (%)		
Central venous line or dialysis central line	12 (57)^c^	NA
Other	8 (38)^d^	NA
Total	13 (62)^e^	NA

Abbreviations: D-dimer, dimerized plasmin fragment D; INR, international normalized ratio; LY30, clot lysis at 30 minutes after maximum clot strength; NA, not applicable.

SI conversion factors: To convert platelet count to ×10^9^ per liter multiply by 1; fibrinogen to grams per liter, multiply by 0.01; and D-dimer to nanomoles per liter, multiply by 5.476.

^a^ Measured at the time of intensive care unit admission.

^b^ Measured after heparinase treatment.

^c^ Includes 22 total events.

^d^ Includes arterial line thrombosis (5 patients [8 events]), dialysis filter failure unassociated with dialysis central line catheter thrombosis (2 patients [3 events]) and arterio-venous fistula thrombosis (1 event).

^e^ Includes 46 events.

There were no statistically significant differences in prothrombin time, INR, partial thromboplastin time, or platelet levels between 10 patients with at least 2 thrombotic events vs 11 patients with fewer than 2 events (Table 2). In comparison, innate TEG MA was significantly greater for the high event rate group than the low event rate group (mean [SD], 75 [7] mm vs 61 [21] mm; *P* = .01). Elevated MA was observed in 10 patients (100%) in the high event rate group vs 5 patients (45%) in the low event rate group. Innate TEG MA provided 100% sensitivity and 100% negative predictive value (Table 2).

**Table 2.** Comparison of Routine and Thromboelastography Coagulation Parameters in Low and High Thrombotic Event Rate Groups

Parameter	Event rate group, mean (SD)	
	Low (0-1 thrombotic events) (n = 11)	High (≥2 thrombotic events) (n = 10)
Prothrombin time, s	14.5 (1.6)	15.1 (3.1)
INR	1.2 (0.1)	1.3 (0.3)
Partial thromboplastin time, s	30.6 (3.4)	32.1 (2.5)
Platelet count, ×10^3^/µL	200 (83)	242 (98)
Fibrinogen level, mg/dL	707 (213)	804 (256)
D-dimer, μg/mL	2.9 (1.8)	6.8 (6.6)
Thromboelastography		
Innate		
R value, min	13 (14)	7.1 (5)
Fibrinogen activity angle, °^a^	52 (27)	68 (16)
Maximum amplitude, mm^b^	61 (21)	75 (7)
LY30, %	1.3 (2.4)	0.5 (0.7)
After heparinase		
R value, min	6.1 (2.6)	5.9 (3)
Fibrinogen activity angle, °	71 (11)	75 (9)
Maximum amplitude, mm	72 (11)	77 (7)
LY30, %	3.5 (4.6)	0.6 (1)
Patients with thrombotic complications, No. (%)		
Central venous line or dialysis central line	1 (5)	10 (48)
Other	2 (10)^c^	7 (29)^d^
Total	3 (14)	10 (48)^e^

Abbreviations: D-dimer, dimerized plasmin fragment D; INR, international normalized ratio; LY30, clot lysis at 30 minutes after maximum clot strength.

SI conversion factors: To convert platelet count to ×10^9^ per liter multiply by 1; fibrinogen to grams per liter, multiply by 0.01; and D-dimer to nanomoles per liter, multiply by 5.476.

^a^ Sensitivity: 70%; specificity: 64%; positive predictive value: 64%; negative predictive value: 70%.

^b^ Sensitivity: 100%; specificity: 55%; positive predictive value: 67%; negative predictive value: 100%.

^c^ Includes 1 arterial line thrombosis and 1 dialysis filter failure.

^d^ Includes 5 arterial line thromboses, 1 dialysis filter failure, and 1 arteriovenous fistula thrombosis.

^e^ Includes 7 patients with thrombotic events in more than 1 category.

## Discussion

This cohort study found that higher thromboses rates were associated with TEG results outside reference ranges among patients with COVID-19 who were critically ill. Risk associated with TEG results outside reference ranges manifested as a 62% thrombosis event rate, 2-fold the thrombosis event rates that have been previously reported, despite our use of recommended deep vein thrombosis prophylaxis.^4,5^ Underdiagnosis or undertreatment of hypercoagulation may explain the high incidence of unexplained COVID-19 mortalities. These may be associated with potentially preventable microvascular and macrovascular thromboses and consequent cardiovascular complications, including myocardial injury and infarction.^5,6^ Accordingly, our institution and other health care systems have adopted immediate full heparinization in patients with high-acuity COVID-19.

Hypercoagulation associated with COVID-19 may be due to increased angiotensin II expression secondary to angiotensin-converting enzyme 2 receptor binding and consequently increased plasminogen activator inhibitor C-1 expression, which is consistent with our observation of reduced fibrinolysis in our high thrombotic event rate group.^5,6^ Similarly, angiotensin II–mediated pulmonary vasoconstriction can lead to stasis and hypercoagulability, as can COVID-19 induction of antiphospholipid antibodies and complement during cytokine storms, causing vasculitis and microthromboses.

Our finding of INR, partial thromboplastin time, and platelet levels within or close to reference ranges but elevated fibrinogen and dimerized plasmin fragment D levels reflect a complex inflammatory and hematologic profile distinct from the disseminated intravascular coagulopathy associated with COVID-19. In this context, TEG may be critical in accurately identifying patients at increased thrombosis risk and thereby avoiding unnecessary anticoagulation in patients with low thrombosis risk. Specifically, a hypercoagulable innate TEG MA yielded 100% sensitivity and 100% negative predictive value for the occurrence of multiple thromboses.

One study limitation is whether this retrospective study reflects differences in our anticoagulation practices vs other institutions or their underreporting of thrombotic events, as recently suggested.^4,5,6^ Our findings suggest that alterations of diagnostic and prophylactic treatment guidelines may be critical for the successful treatment of coagulopathies associated with COVID-19.

## References

[1] Han H, Yang L, Liu R, Prominent changes in blood coagulation of patients with SARS-CoV-2 infection. Clin Chem Lab Med. Published online March 16, 2020;/j/cclm.ahead-of-print/cclm-2020-0188/cclm-2020-0188.xml. doi:10.1515/cclm-2020-0188 32172226

[2] Tang N, Bai H, Chen X, Gong J, Li D, Sun Z Anticoagulant treatment is associated with decreased mortality in severe coronavirus disease 2019 patients with coagulopathy. J Thromb Haemost. 2020;18(5):1094-1099. doi:10.1111/jth.14817 32220112 PMC9906401

[3] Thachil J, Tang N, Gando S, ISTH interim guidance on recognition and management of coagulopathy in COVID-19. J Thromb Haemost. 2020;18(5):1023-1026. doi:10.1111/jth.14810 32338827 PMC9906133

[4] Geerts W Central venous catheter-related thrombosis. Hematology Am Soc Hematol Educ Program. 2014;2014(1):306-311. doi:10.1182/asheducation-2014.1.306 25696870

[5] Klok FA, Kruip MJHA, van der Meer NJM, Incidence of thrombotic complications in critically ill ICU patients with COVID-19. Thromb Res. 2020;(April):S0049-3848(20)30120-1. doi:10.1016/j.thromres.2020.04.013 32291094 PMC7146714

[6] Dolhnikoff M, Duarte-Neto AN, de Almeida Monteiro RA, Pathological evidence of pulmonary thrombotic phenomena in severe COVID-19. J Thromb Haemost. Published online April 15, 2020. doi:10.1111/jth.14844 32294295 PMC7262093

